# Paeonol Inhibits Oxidized Low-Density Lipoprotein-Induced Vascular Endothelial Cells Autophagy by Upregulating the Expression of miRNA-30a

**DOI:** 10.3389/fphar.2018.00095

**Published:** 2018-02-08

**Authors:** Chao Li, Li Yang, Hongfei Wu, Min Dai

**Affiliations:** ^1^School of Pharmacy, Anhui University of Chinese Medicine, Hefei, China; ^2^Key Laboratory of Xin’an Medicine, Ministry of Education, Hefei, China; ^3^Anhui Key Laboratory for Research and Development of Traditional Chinese Medicine, Hefei, China

**Keywords:** paeonol, oxidized low density lipoprotein, autophagy, microRNA-30a, vascular endothelial cell

## Abstract

Paeonol from Cortex Moutan root is a potential therapeutic agent for atherosclerosis (AS). However, its mechanisms of action are still not fully understood. Vascular endothelial cells (VECs) autophagy plays a vital role in the initiation and progression of AS. In this study, we aim to investigate whether the protective effect of paeonol on ox-LDL-induced VECs injury by regulating autophagy. To address this question, we used ox-LDL-induced rat VECs as a model system to elucidate the protective effect of paeonol on VECs injury. This study displayed that ox-LDL (100 mg/L) treatment inhibited VEC growth in dose- and time-dependent manners, paeonol (60 μM) shown potential in inhibiting ox-LDL-induced death. Furthermore, paeonol significantly reduced ox-LDL-induced the formation of autophagy vacuoles and the expression of LC3II in VECs. Further double-luciferase reporter assay shown that miR-30a specifically binds to the 3′-UTR of Beclin-1 mRNA in VECs. Moreover, we found that ox-LDL decreased miR-30a and increased Beclin-1 expression, pretreatment with paeonol could reverse the process of regulation in dose-dependent manners. In ox-LDL treated VECs, transfection with a miR-30a mimic significantly increased miR-30a expression and inhibited Beclin-1 and LC3II expression, thus enhanced the protective effects of paeonol. Whereas transfection with a miR-30a inhibitor significantly decreased miR-30a expression and increased Beclin-1 and LC3II expression, thus attenuated the protective effects of paeonol. In conclusion, this study has, for the ?rst time, highlighted that miR-30a might be a critical target of paeonol against ox-LDL-induced VECs injury by inhibiting excessive autophagy. Paeonol may be one of promising candidate drug for treatment of AS.

## Introduction

Atherosclerosis (AS) is featured as a chronic inflammatory disease of vascular stenosis due to lipid per-oxidation, release of inflammatory mediators by activated macrophage and vascular endothelial cells (VECs) dysfunction (Ross, 1995; Forstermann et al., 2017). Paeonol (2′-hydroxy-4′-methoxyacetophenone, C_9_H_10_O_3_) is a natural phenolic compounds isolated from a traditional Chinese medicine, Cortex Moutan, which exhibits anti-AS effects *in vitro and in vivo*. Moreover, further studies demonstrate that it modulates a variety of physiological and pathological processes involved in anti-thrombotic, antioxidant properties, and anti-inflammatory (Fu et al., 2012; Bao et al., 2013). Our previous investigations have found its anti-AS effects are closely related to protect against ox-LDL-induced the damage of VECs by regulating miR-21 and miR-126 (Li et al., 2009; Liu et al., 2014; Yuan et al., 2016).

Several lines of evidence indicated that the autophagy of VECs was closely related to the major diseases such as AS and other vascular diseases. Recent studies have shown that an elevated autophagy was beneficial for cell to adapt to the different pathological conditions (Klionsky and Codogno, 2013; Fulda and Kögel, 2015). On the contrary, autophagy also promotes autophagic cell death through excessive self-digestion and degradation of essential cellular constituents. Nevertheless, it remains uncertain whether the mechanisms underlying autophagy activation have any influence on the participation of VECs in atherosclerotic plaque formation (Luo et al., 2016). Recent studies have shown that autophagy of VECs can be regulated by a range of biological factors and chemical compounds (Jiang, 2016).

MicroRNAs (miRNAs or miRs) are a group of small molecules that can regulate gene expression, and play a vital role in many pathophysiologic processes such as endothelial injury (Qin and Zhang, 2011; Aryal et al., 2017; Njock and Fish, 2017). Beclin-1 is a pivotal autophagy-promoting gene for regulating death and survival of multifarious categories of cells (Zhu et al., 2009). We noticed that the miR-30a might modulate autophagic response by targeting the autophagy-promoting gene Beclin-1 expression (Long et al., 2013). Nevertheless, there is no report on the links between the function of paeonol and miRNAs in regard to autophagy currently. Therefore, we were especially interested in determining whether paeonol effectively regulating target protein expression of Beclin-1, thus affecting autophagy by modulating miR-30a in VECs.

In this study, we investigated the mechanisms underlying the protective effect of paeonol on VECs injury model. We further explored the biological function of miR-30a in the presence or absence of paeonol treatment in ox-LDL-injured VECs. Collectively, paeonol may protect VECs against ox-LDL-induced autophagy by modulating miR-30a. Thus, miR-30a may be a potential therapeutic target for treatment of AS with the use of paeonol.

## Materials and Methods

### Ethics Statement

All surgical and experimental procedures were approved by the Ethics Review Committee for Animal Experimentation of the Institute of Clinical Pharmacology at Anhui Medical University.

### Animals

Healthy male Sprague-Dawley rats weighing 140–170 g were provided by the Experimental Animal Center of Anhui Medical University (Hefei, China). The animal experiments were approved by the local institutional Animal Care and Use Committee.

### Chemicals and Reagents

We purchased paeonol (99% purity) from Baicao Plants Biotech, Co., Ltd. (Anhui, China). Ox-LDL (Cat. YB-002-1) was purchased from Yiyuan Biotechnologies, Co., Ltd. (Hangzhou, China). The BCA Protein Assay kit was obtained from Shanghai Haoran Bio Technologies, Co., Ltd. (China). HiPerFect transfection reagent (Cat. 301704), MiRNeasy Mini Kit (Cat. 217004), and MiScript PCR Starter Kit (Cat. 218193) were obtained from Qiagen (Germany). miR-30a inhibitor and mimic were purchased from zoonbio Biotechnology, Co., Ltd. (Nanjing, China). The dual-luciferase reporter system (Cat. E1910) was purchased from Promega (United States). Antibodies against β-actin (Cat. BS6007M), Beclin-1 (Cat. AP0769), and LC3 (Cat. AP1013) were purchased from Bioworld Technology, Co., Ltd. (United States).

### Isolation and Culture of VECs

Vascular endothelial cells were isolated from Sprague-Dawley rats thoracic aortas as previously described (Pan et al., 2007). In short, thoracic aortas of rats turned over to expose the luminal surface, under aseptic condition. Suture line tied tightly the both ends of the aorta and digested it with 0.2% collagenase I (Sigma, United States), incubation at 37°C in 5% CO_2_ for 1 h, the aortas were washed with medium, then, cut into pieces and placed luminal side down onto collagen-coated flask containing DMEM supplemented with 15% fetal bovine serum (FBS, Gibco, United States), 2 mM L-glutamine (Sigma), 1% penicillin and streptomycin (Gibco) and then incubated at 37°C with 5% CO_2_. After 4–6 days, the explants were removed, when the cells had formed a monolayer, and the cells were subcultured. VECs should be released from their substrate by trypsinization. Cobblestone like cell morphology and the purity of the VECs was determined by immunocytochemical staining for Factor VIII (Bioss, Beijing, China) were identified by the VEC-specific. In all experiments, VECs used were passage 4.

### MTT Assay

Vascular endothelial cells (1 × 10^5^ cells/mL) were seeded in 96-well-plates and incubated in DMEM with 15% FBS incubate the cells under conditions appropriate for the cell line for 6 h at 37°C in 5% CO_2_. After treatment with serially diluted concentrations of paeonol and ox-LDL, add 20 μL of MTT (5 mg/mL) Reagent to each well, including controls, and then the incubations were continued for another 4 h. When the purple precipitate is clearly visible under the microscope, the medium was then removed, and 200 μL DMSO was added to each well to dissolve the precipitate at 570 nm in a microtiter plate reader (Spectra Max Mze, St. Albans, VT, United States).

### Electron Microscopy

Vascular endothelial cells were pretreated with liquid medium contain different concentration of paeonol (60 μM) for 24 h. The culture medium was sucked out, PBS cleaning. And then liquid medium contain ox-LDL (100 mg/L) treat for 24 h and then the cells were collected, 1,000 rpm centrifuge 5 min, the supernatant was discarded, 1 mL PBS resuspended, centrifuged 10 min, the supernatant was discarded, fixed with 2.5% glutaraldehyde, and then the sample were sent to the testing organizations. After dehydration in a graded series of acetone, the cells were embedded in spur resin. Thin sections were cut on a Reichert Ultracut E microtome. Sectioned grids were stained with saturated solution of uranyl acetate and lead citrate. Sections were examined at 80 kV with a JEOL 1200EX transmission electron microscope.

### Western Blotting

Vascular endothelial cells were washed and scrapped in PBS, then disrupted in RIPA lysis buffer at 4°C for 30 min on ice. Cell extracts were centrifuged at 12,000 × *g* for 15 min and the supernatant was used for Western blotting experiments. Protein concentration was determined using the BCA Protein Assay kit. The proteins were separated by 10% SDS-polyacrylamide gel electrophoresis and transferred onto PVDF membranes. Then, membranes were incubated with antibodies against β-actin and Beclin-1 and revealed with secondary antibodies coupled to horseradish peroxidase using the ECL chemoluminescence kit.

### RNA Extraction and qRT-PCR Assay

Vascular endothelial cells (1 × 10^5^ cells/mL) were inoculated in 6-well-plates cultured in cell incubator incubated in a standby. The configuration of transfection complex: miR-30a mimic, miR-30a inhibitor (20 μM) respectively 0.6 and 12 μL HiPerFect transfection reagent mixed by vortexing to form transfection complexes was added to the 400 μL without serum or antibiotics DMEM medium, 5–10 min transfection complexes at room temperature will be uniform into VECs cell culture medium. Then the cells were cultured for 24 h at 37°C in 5% CO_2_, then replaced with the Paeonol and ox-LDL medium according to the experimental purpose.

Total RNA was isolated using QIAzol Lysis Reagent and miRNeasy Mini Kits according to the manufacturer’s protocol for both miR-30a and Beclin-1 mRNA analyses. For detection of gene expression, qRT-PCR was performed using Quantitect SYBR Green PCR Kits according to the manufacturer’s instructions. Gene expression levels were normalized to those of β-actin or U6 for Beclin-1 mRNA and miR-30a, respectively. The following primers (Sangon, China) were used: Beclin-1, forward 5′-TTC AAG ATG GAC CGA GTG AC-3′, reverse 5′-AGA CAC CAT CCT GGC GAG TTT C-3′; β-actin, forward 5′-GAT TAC TGC CCT GGC TCC TA-3′, reverse 5′-TCA TCG TAC TCC TGC TTG CT-3′; miR-30a, forward 5′-GAC GGT ACC TGG TGG AGA ACA ACT TCG-3′, reverse 5′-CAG AAG CTT CAT CAA ACC TTC AAT CCC-3′; U6,-forward 5′-GCT TCG GCA GCA CAT ATA CTA AAA T-3′, reverse 5′-CGC TTC ACG AAT TTG CGT GTC AT-3′. Changes in gene expression were determined using the 2^-ΔΔCt^ method.

### Dual-Luciferase Reporter Assay

The wild type (WT) 3′-UTR of human Beclin-1 (5′-GCA AGC CAG ACA GGA AAA AG-3′) and the 3′-UTR sequences carrying mutations (5′-CCT TAA CGA AAA TTT CCT ATG TCT GTC TAT TGG TAT GC-3′) were amplified and subcloned into the pGL3 expression vector downstream of the promoter and coding region of firefly luciferase (Promega, United States). The sequence for miR-30a was obtained from bioinformatics web sites, miTarget, and the matched sites are shown below. VECs (1 × 10^5^ cells/mL) were seeded in 24-well-plates, and each transfected with 100 ng of pGL3-Beclin-1-3′-UTR and pGL3-Beclin-1-3′-URT-mut and miR-30a mimics or inhibitor using HiPerFect transfection reagent. After 36 h, luciferase activity was measured by using the dual-luciferase assay system.

### Statistical Analysis

Data were expressed as the mean ± SEM and analyzed using SPSS version 21 statistical analysis package (SPSS, Inc., United States). Student’s *t*-test was used for statistical comparisons between two groups. For comparison of more than two groups, one-way ANOVA was used for multiple groups. Statistical significance was considered at *P* < 0.05.

## Results

### Paeonol Attenuates Autophagy in ox-LDL-Induced VECs

**FIGURE 1:**
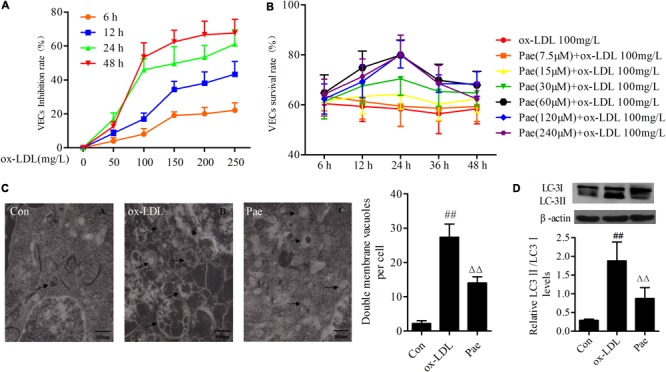
Paeonol attenuates function of autophagy in ox-LDL-stimulated VECs. **(A)** The VECs were treated with serially diluted ox-LDL (50, 100, 150, 200, 250 mg/L) for different periods (6, 12, 24, 48 h) and subsequently the inhibitory rate as evaluate with the MTT assay. And it has a time- and dose-response relationship suppressed the growth of VECs. **(B)** VECs viability was detected by MTT assay after pretreatment with increasing concentrations of paeonol (7.5, 15, 30, 60, 120, 240 μM) for different period (6, 12, 24, 48 h), then treated with 100 mg/L ox-LDL for another 24 h. **(C)** Rat VECs autophagy by Electron Microscope (× 15000), arrows indicate autophagic vacuoles. Each sample counts six cells and showing the average number of double membrane vacuoles per cell. The data were expressed as mean ± SEM, *n* = 6. **(D)** Western blotting showing the expression of LC3 protein in VECs. Data were expressed as mean ± SEM, *n* = 3. ANOVA testing was performed; ^##^*P* < 0.01 vs. control; ^ΔΔ^*P* < 0.01 vs. ox-LDL group.

Vascular endothelial cells survival rates were examined by the MTT assay after treatment with increasing concentrations of ox-LDL (50, 100, 150, 200, 250 mg/L) at different periods (6, 12, 24, or 48 h). Ox-LDL caused VECs death as compared with the control group in dose-/time-dependent manner; when ox-LDL (100 mg/L) treated with 24 h, it significantly inhibited the growth of VECs (**Figure 1A**). Based on the MTT results, we tried to establish optimal concentrations and action time of paeonol in VECs. VECs were pretreated with increasing concentrations of paeonol (7.5, 15, 30, 60, 120, 240 μM) for different time (6, 12, 24, or 48 h). Ox-LDL (100 mg/L) were cultured with VECs for another 24 h. Compared with ox-LDL group, paeonol could significantly increase the VECs survival rate in dose- and time-dependent manner (**Figure 1B**). An optimal condition for paeonol treatment in VECs (60 μM for 24 h) was established and used in subsequent experiments.

Previous studies shown that ox-LDL could induce autophagy. To investigate whether autophagy was involved in VECs dysfunction under ox-LDL-induced state, we examined the procedure of autophagy by electron microscopy, arrows indicate autophagic vacuoles. Ox-LDL upregulated the formation of autophagic vacuoles in VECs for 24 h, However, paeonol suppressed formation of autophagic vacuoles (*P* < 0.01) (**Figure 1C**). To further confirm whether paeonol could protecte cells against ox-LDL-induced autophagy, the expression of LC3 protein was measured. And as expected, Ox-LDL remarkably increased the levels of LC3II protein, and pretreatment with paeonol reduced the levels of LC3II protein in VECs, which demonstrates that paeonol has an inhibitory effect on oxLDL-induced VECs autophagy (*P* < 0.01) (**Figure 1D**).

### miR-30a Prevents Upregulation of Beclin-1 in ox-LDL-Stimulated VECs

**FIGURE 2:**
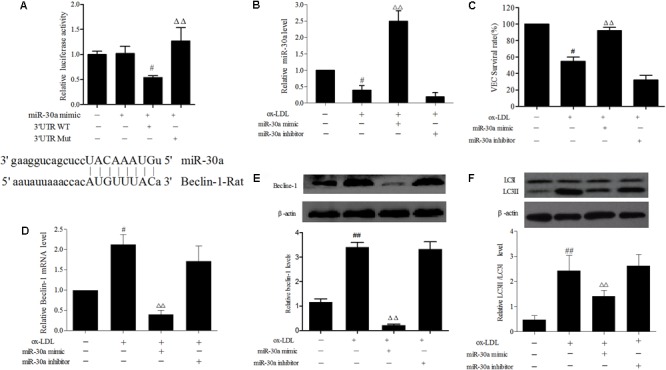
miR-30a prevents upregulation of Beclin-1 in ox-LDL-stimulated VECs. **(A)** Dual-luciferase gene reports experiments used to verify Beclin-1 was target gene of miR-30a in VECs. The data were expressed as mean ± SEM, *n* = 3. ANOVA testing was performed; ^#^*P* < 0.05 vs. miR-30a mimic group. ^ΔΔ^*P* < 0.01 vs. miR-30a mimic and 3′ UTR wild type (WT) group. **(B)** The levels of miR-30a were detected by SYBR Green PCR. VECs were transfected with miR-30a mimic and inhibitor, after ox-LDL-stimulated VECs for 24 h, then expression of miR-30a was measured by SYBR Green PCR. **(C)** VECs viability was detected by MTT. **(D–F)** SYBR Green PCR showing the expression of Beclin-1 mRNA in VECs and western blotting showing the expression of Beclin-1, LC3 protein in VECs. MiR-30a mimic or inhibitor was transfected into VECs, after ox-LDL-stimulated VECs for 24 h. Data were expressed as mean ± SEM, *n* = 3. ANOVA testing was performed; ^##^*P* < 0.01, ^#^*P* < 0.05; vs. control group. ^ΔΔ^*P* < 0.01 vs. ox-LDL group.

In order to determine biological role underlying the interaction between miR-30a and Beclin-1 in VECs, the dual luciferase reporter system shown that miR-30a mimic acting on Beclin-1 mRNA WT 3′-UTR plasmid, obviously reduced the firefly luciferase activity (*P* < 0.05), whereas cotransfection with mutant type 3′-UTR plasmid elicited significantly higher luciferase activity than that seen in the WT group (*P* < 0.01) (**Figure 2A**). It was clearly demonstrated that Beclin-1 served as a target gene of miR-30a in the VECs.

In addition, to ulteriorly explain the relationship between miR-30a and Beclin-1 in ox-LDL treated VECs, the role of miR-30a was transfect with a miR-30a mimic which markedly enhanced miR-30a levels in ox-LDL-induced VECs, whereas miR-30a inhibitor significantly suppressed miR-30a expression (*P* < 0.01) (**Figure 2B**). As expected, compared with the ox-LDL group, VECs survival rate was increased in miR-30a mimic (*P* < 0.01) and that these changes were reversed by treatment with the miR-30a inhibitor (**Figure 2C**). Overexpression of miR-30a strikingly reversed the expression of Beclin-1 at both the mRNA and protein in ox-LDL treated VECs. Consistently, the insignificant effect of miR-30a inhibitor to enhance the miR-30a and Beclin-1 expression in ox-LDL-induced VECs (**Figures 2D,E**). Therefore, these findings suggested that Beclin-1 3′-UTR was directly regulated by miR-30a at the post-transcriptional level in VECs. To further examine whether ox-LDL regulated autophagy via the miR-30a. Our results shown that LC3II protein has a remarkably low expression in the miR-30a mimic group compared with the ox-LDL group (*P* < 0.01) (**Figure 2F**).

### Paeonol Increases miR-30a Levels in ox-LDL-Induced VECs

**FIGURE 3:**
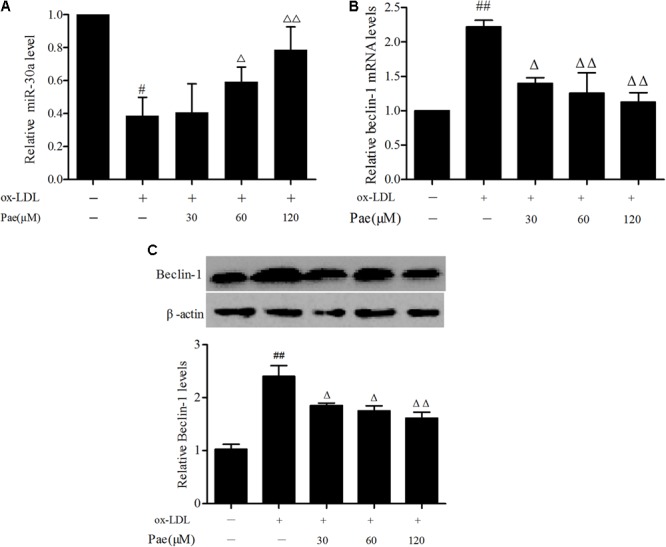
Paeonol increases miR-30a levels in ox-LDL-stimulated VECs. **(A)** The levels of miR-30a in VECs ox-LDL were detected by SYBR Green PCR. VECs pretreated with different concentrations of paeonol (30, 60, 120 μM) for 24 h, and then the VECs during their treatment with liquid medium contain ox-LDL (100 mg/L) for another 24 h **(B,C)**. Western blotting showing the expression of Beclin-1 protein and SYBR Green PCR showing the expression of Beclin-1 mRNA in VECs. Values are expressed as mean ± SEM, *n* = 3. ANOVA testing was performed; ^#^*P* < 0.05, ^##^*P* < 0.01 vs. control. ^Δ^*P* < 0.05; ^ΔΔ^*P* < 0.01 vs. ox-LDL group.

MiR-30a as a crucial regulator of autophagy has been reported in extensive studies (Zhu et al., 2009). In order to explore the protective effect of paeonol was related to the change of miR-30a. VECs were pretreated with paeonol (30, 60, 120 μM) for 24 h and co-cultured with ox-LDL for another 24 h. Total RNA in each group were extracted. qRT-PCR assay was used to detect relative miR-30a levels. The results shown that ox-LDL could decrease miR-30a levels (*P* < 0.01), as compared with the control group. However, paeonol gradually increased the expression of miR-30a in a dose-dependent manner (*P* < 0.05) (**Figure 3A**), suggesting that the protective effect of paeonol on VECs was associated with modulation of miR-30a expression.

To test whether the protective effect of paeonol in ox-LDL-treated VECs was associated with regulation of Beclin-1, we treated VECs with paeonol (30, 60, 120 μM) for 24 h, followed by 24 h of ox-LDL treatment. Ox-LDL treatment significantly suppressed the expression of Beclin-1 at both the mRNA and protein, and these effects were prevented by pretreatment with paeonol in dose-dependent manner (*P* < 0.05) (**Figures 3B,C**).

### Paeonol Attenuates ox-LDL-Induced VECs Autophagy by Upregulating the Expression of miR-30a

**FIGURE 4:**
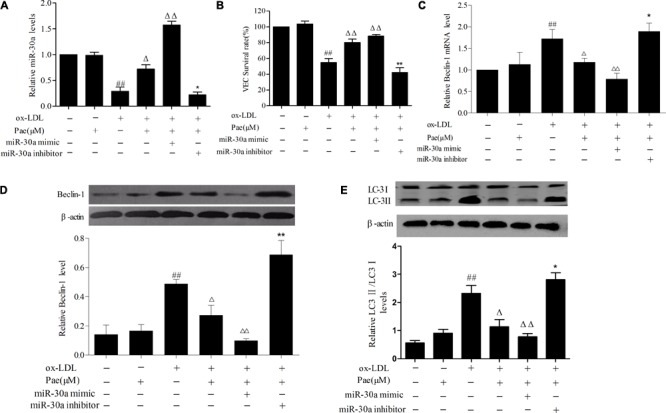
Paeonol increases miR-30a levels in VECs stimulated with ox-LDL. **(A)** The levels of miR-30a in VECs ox-LDL were detected by SYBR Green PCR. VECs were transfected with miR-30a mimic and inhibitor, after paeonol pretreatment with 60 μM for 24 h and ox-LDL-stimulated VECs for another 24 h, then expression of miR-30a was measured by SYBR Green PCR. **(B)** VECs viability was detected by MTT assay after transfected with miR-30a mimic and inhibitor. **(C–E)** SYBR Green PCR showing the expression of Beclin-1 mRNA in VECs and western blotting showing the expression of Beclin-1, LC3 protein in VECs. MiR-30a mimic or inhibitor was transfected into VECs, after paeonol pretreatment with 60 μM for 24 h and ox-LDL-stimulated VECs for another 24 h. Data were expressed as mean ± SEM, *n* = 3. ANOVA testing was performed; ^##^*P* < 0.01 vs. control group. ^ΔΔ^*P* < 0.01; ^Δ^*P* < 0.05 vs. ox-LDL group. ^∗∗^*P* < 0.01; ^∗^*P* < 0.05 vs. ox-LDL and paeonol group.

Furthermore, in order to further confirm the role of paeonol in the modulation the relationship between miR-30a and Beclin-1 in ox-LDL-induced VEC, we found that paeonol counteracted the effect of transfected with miR-30a inhibitor in VECs, decreasing miR-30a levels (*P* < 0.01) (**Figure 4A**). We observed a higher increase of Beclin-1 and LC3II levels than pretreatment with paeonol ox-LDL-induced VECs (*P* < 0.01). The effect of paeonol was enhanced by transfection with the miR-30a mimic, however, was significantly eliminated by miR-30a inhibitor (*P* < 0.01). These results suggested that paeonol might regulate Beclin-1 by miR-30a to protect VECs from ox-LDL-induced VECs injury (**Figures 4B–E**).

## Discussion

In this study, we have shown that paeonol, the main active compound of the traditionally used Chinese herb from *Paeonia Suffruticosa* Andrews, has VECs protective activities. A model of ox-LDL-impaired VECs has been applied to mimic the oxidative endothelial injury that occurs during atherogenesis (Wu et al., 2016). Paeonol has protective effect on VECs exposed to ox-LDL by increasing VECs growth. Numerous studies have shown that ox-LDL increased the LC3II and Beclin-1 levels in VECs (Nowicki et al., 2007). Autophagy was found in ox-LDL-induced VECs damage, these results are entirely consistent with our studies. Surprisingly, we found that pretreatment with paeonol could reverse the process of regulation. Further analysis shown that miR-30a directly binds to the 3′-UTR of Beclin-1 mRNA in VECs and miR-30a mimic significantly increased miR-30a expression and inhibited Beclin-1 and LC3II expression. However, paeonol gradually increased the expression of miR-30a in a dose-dependent manner. Moreover, miR-30a inhibitor significantly decreased miR-30a expression and increased Beclin-1 and LC3II expression, thus attenuated the protective effects of paeonol.

The role of autophagy in different diseases are diverse and could promote different outcomes even in a single cell, thus its mechanism remains unclear. The present research has indicated autophagy of VECs is considered as a stage-dependent dual player in AS (Shintani and Klionsky, 2004; Levine and Yuan, 2005; Li et al., 2008). Under stress conditions of oxidant injury, cell starvation, hypoxia, and other damaging insults, autophagy activation tends to increase VECs survival (Klionsky and Codogno, 2013). In most instances, timely and appropriate autophagy could inhibit the accumulation of ox-LDL in VECs and the abnormal secretion of VECs inflammatory factors, and ensure intracellular homeostasis (Fulda and Kögel, 2015). However, in some instances, excessive autophagy leads to dysregulation of major proteins in VECs, thereby might contribute to the VECs injury and death (Chau et al., 2003; Green and Levine, 2014; Gatica et al., 2015). More interestingly, VECs challenged with hypoxia, there is a transition from autophagy-mediated cell survival to autophagy-mediated cell death in a time-dependent manner (Ochi et al., 2015). The beneficial effects of autophagy in VECs are likely to be context-dependent, since autophagy may also contribute to cell death under certain circumstances. These discrepancies may be because of the different types of VECs used, the different passages of cells, the different types of autophagy analyzed, as well as the different ox-LDL concentration and time used. In our study, we found ox-LDL could induce autophagy and inhibit cells survival, indicating that ox-LDL may cause excessive autophagy in VECs, as the same time, we found paeonol could reduce the formation of autophagic bubbles, downregulate autophagy related proteins LC3II levels and reduce ox-LDL-induced VECs death. We hold the notion that paeonol anti-AS may be related to the regulation of autophagy in VECs.

MiRNA, as a small non-coding RNA, has a specific expression in cardiovascular disease and plays an important regulatory role to regulate gene expression. Zhu et al. (2009) found that miR-30a could suppress autophagy by negatively regulating its target gene–Beclin-1 that is a homolog of yeast autophagy gene ATG6/VPS30 and a positive regulator of autophagy. Furthermore, to determine the link between downregulation of miR-30a and activation of autophagy, we have used many methods. First of all, the miRNA target gene prediction software and some related literatures indicated that it had a strong correlation between the 3′-UTR of Beclin-1 mRNA and miR-30a. Secondly, double luciferase gene reporter also indicated that miR-30a suppresses Beclin-1 by directly binding to its 3′-UTR. Therefore, Our results, as described in other reports (Zhu et al., 2009), shown that the direct target of miR-30a is Beclin-1 mRNA in VECs. Lastly, we further investigated miR-30a expression in ox-LDL-induced VECs by transfected miR-30a minic and inhibitor. As expected, we observed that ox-LDL downregulates miR-30a expression and upregulates Beclin-1 and LC3II expression in VECs. Our results shown that Beclin-1 and LC3II had a remarkably lower expression in the miR-30a mimic group and the insignificant effect of miR-30a inhibitor to enhance the injury of ox-LDL, which suggested that miR-30a regulated Beclin-1 protein and might be a suppressor for autophagy.

In order to test whether the protective effect of paeonol on ox-LDL-induced injury in VECs were associated with changes in miR-30a expression, the expression of miR-30a after paeonol and ox-LDL treatment was measured by qRT-PCR. As expected, Paeonol gradually increased the expression of miR-30a and reduced the Beclin-1 expression in a dose-dependent manner. However, we found that protective effect of paeonol on ox-LDL-induced injury was completely ineffective through intervention of miR-30a inhibitor. Obviously, paeonol representing a possible regulator of miR-30a expression, this indicated that protective effect of paeonol was related to modulation of miR-30a levels.

## Conclusion

In summary, our current results showed that paeonol could downregulate the autophagy in ox-LDL-treated VECs by increasing miR-30a levels and reducing the expression of Beclin-1 protein, which suggested miR-30a could be a potential target for AS treatment. Nevertheless, further investigation is warranted for the therapeutic potential of paeonol in AS treatment.

## Author Contributions

CL wrote the paper; CL and LY performed the experiments and prepared the figures. CL and HW analyzed and commented the results. MD designed the study and supervised the project. All authors have read and approved the final manuscript.

## Conflict of Interest Statement

The authors declare that the research was conducted in the absence of any commercial or financial relationships that could be construed as a potential conflict of interest.
